# Liver targeting with rAAV7: balancing tropism with immune profiles

**DOI:** 10.1038/s41434-021-00230-4

**Published:** 2021-02-05

**Authors:** Terence R. Flotte

**Affiliations:** University of Massachusetts Medical School, Worcester, MA USA

**Keywords:** Genetics, Gene regulation

The value of intravenous (IV) recombinant adeno-associated virus (rAAV) as a platform for delivery of transgenes to the liver for gene therapy has been well established. The most notable example is the use of a self-complementary rAAV9 vector expressing a high specific activity Factor IX gene in patients with hemophilia B [1–3]. Since the liver is the primary site for expression of numerous other serum proteins and biochemical pathways, this platform has the potential for very broad utility in a wide variety of genetic diseases. Thus, the optimization of the delivery of rAAV to hepatocytes is of central importance in the field of human gene therapy.

The feasibility of using rAAV by the IV route was dramatically improved when Gao et al. demonstrated the biodiversity of wild-type AAV within human and non-human primate populations [4, 5]. A number of the novel rAAV serotypes, such as AAV8 and AAV9, were found to have a strong liver tropism in mouse and non-human primate models [6]. These serotypes were advanced to the clinic as therapy for a wide range of genetic diseases, including the hemophilia B trials referenced above. Interestingly, murine liver has not been faithfully predictive of capsid tropism in larger animals or humans, leading to the use of both non-human primate studies and studies in human hepatocyte xenografts in mice to select serotypes for clinical product development [7, 8].

The work described in the paper by Shao et al. [9] is based on the concept that the selection of an AAV serotype for liver-directed human gene therapy should be determined by the combination of maximal hepatocyte tropism and a minimum level of pre-existing anti-capsid immunity [10]. In this paper, Shao et al. [9] compare rAAV serotypes in a humanized liver xenograft model and demonstrate that rAAV7 is actually superior in hepatocyte tropism to either rAAV8 or rAAV3B, as determined with hepatocytes from multiple different individual human donors. Furthermore, a survey of seropositivity in the general population indicated that pre-existing anti-capsid immunity is less prevalent to AAV7 than to either AAV8 or AAV3B. Surprisingly, however, anti-AAV7 immunity is more prevalent in the hemophilia B population than with either of these two serotypes.

Based on these findings, rAAV7 could be a very useful vector for human gene therapy of diseases other than hemophilia B, but less so for hemophilia B patients. This discovery is somewhat puzzling. One might expect seroprevalence to be independent of disease diagnosis. However, the observations by Shao et al., prompt questions as to whether the health care environment to which hemophilia B patients are exposed might predispose them to wild-type AAV7 infections. The broader lesson to be learned may well be that each individual disease population or region could have its own unique profile of AAV capsid immunity. In spite of this, the finding that AAV7 capsid tropism is particularly high for human liver would seem to warrant specific AAV7 screening within each disease population for which hepatocyte transduction may be necessary, particularly as human translation is approached and final decisions are made regarding the composition of the specific capsid-promoter-transgene combination that is optimal for use in human clinical trials. This type of immune profile tailoring could potentially add a new dimension to the concept of precision medicine for human genetic disorders, such that immune status intersects orthogonally with genotype in the selection of the personalized gene therapy for any given patient.
